# Profiling of miRNAs and their interfering targets in peripheral blood mononuclear cells from patients with chronic myeloid leukaemia

**DOI:** 10.3389/fonc.2023.1173970

**Published:** 2023-07-05

**Authors:** Sheng-Cheng Wu, Shiue-Wei Lai, Xin-Jie Lu, Hsing-Fan Lai, Yu-Guang Chen, Po-Huang Chen, Ching-Liang Ho, Yi-Ying Wu, Yi-Lin Chiu

**Affiliations:** ^1^ Division of Hematology and Oncology, Department of Internal Medicine, Tri-Service General Hospital Penghu Branch, Magong City, Taiwan; ^2^ Division of Hematology and Oncology, Department of Internal Medicine, Tri-Service General Hospital, National Defense Medical Center, Taipei City, Taiwan; ^3^ Department of Biochemistry, National Defense Medical Center, Taipei City, Taiwan

**Keywords:** chronic myeloid leukemia, micro RNA, peripheral blood mononuclear cell, apoptosis, hematopoietic stem cell differentiation

## Abstract

**Introduction:**

MicroRNAs may be implicated in the acquisition of drug resistance in chronic myeloid leukemia as they regulate the expression of not only BCR-ABL1 but also genes associated with the activation of drug transfer proteins or essential signaling pathways.

**Methods:**

To understand the impact of specifically expressed miRNAs in chronic myeloid leukemia and their target genes, we collected peripheral blood mononuclear cells (PBMC) from patients diagnosed with chronic myeloid leukemia (CML) and healthy donors to determine whole miRNA expression by small RNA sequencing and screened out 31 differentially expressed microRNAs (DE-miRNAs) with high expression. With the utilization of miRNA set enrichment analysis tools, we present here a comprehensive analysis of the relevance of DE-miRNAs to disease and biological function. Furthermore, the literature-based miRNA-target gene database was used to analyze the overall target genes of the DE-miRNAs and to define their associated biological responses. We further integrated DE-miRNA target genes to identify CML miRNA targeted gene signature singscore (CMTGSS) and used gene-set enrichment analysis (GSEA) to analyze the correlation between CMTGSS and Hallmark gene-sets in PBMC samples from clinical CML patients. Finally, the association of CMTGSS stratification with multiple CML cell lineage gene sets was validated in PBMC samples from CML patients using GSEA.

**Results:**

Although individual miRNAs have been reported to have varying degrees of impact on CML, overall, our results show that abnormally upregulated miRNAs are associated with apoptosis and aberrantly downregulated miRNAs are associated with cell cycle. The clinical database shows that our defined DE-miRNAs are associated with the prognosis of CML patients. CMTGSS-based stratification analysis presented a tendency for miRNAs to affect cell differentiation in the blood microenvironment.

**Conclusion:**

Collectively, this study defined differentially expressed miRNAs by miRNA sequencing from clinical samples and comprehensively analyzed the biological functions of the differential miRNAs in association with the target genes. The analysis of the enrichment of specific myeloid differentiated cells and immune cells also suggests the magnitude and potential targets of differentially expressed miRNAs in the clinical setting. It helps us to make links between the different results obtained from the multi-faceted studies to provide more potential research directions.

## Introduction

Chronic myeloid leukemia (CML) is a myeloproliferative hematological cancer involving hematopoietic stem cells with an incidence of 1-2 cases per 100,000 adults (1). The clinical hallmark of CML is the uncontrolled production of mature or immature granulocytes, mainly neutrophils, but also eosinophils and basophils, with varying patterns of abnormal platelet function (2). The pathogenesis of CML is based on the fusion of the Abelson murine leukemia (ABL1) gene on chromosome 9 with the breakpoint cluster region (BCR) gene on chromosome 22, resulting in the expression of an oncoprotein known as BCR-ABL1 (3). BCR-ABL1 is a combinatorically active tyrosine kinase that promotes proliferation and chromosomal replication through multiple downstream signaling pathways and affects leukemia development by creating a cytokine-independent cell cycle with abnormal apoptotic signals in response to cytokine withdrawal (4–8). The development of small molecule tyrosine kinase inhibitors (TKI), which effectively interfere with the interaction between the BCR-ABL1 oncoprotein and adenosine triphosphate (ATP) and thus block the proliferation of malignant granulocytes, has revolutionized the field of CML therapy. This ‘targeted’ approach has changed the history of CML treatment, raising the 10-year survival rate from around 20% to 80%-90% (9). TKIs for BCR-ABL1 become the current standard of care for patients with chronic phase CML. However, with the widespread use of commercially available TKI and the increasing prevalence of CML, an increasing number of patients are suffering from drug resistance. The direct mechanism of resistance involves point mutations in the structural domain of the BCR-ABL1 kinase, which undermines the activity of the available TKI. Although second-generation TKI have overcome most mutations that were resistant to imatinib, new mutations have emerged that make leukemia resistant to them (10). More so, the role of soluble cytokines, drug transfer proteins, micro vesicles and the impact of inflammation and immune surveillance on microenvironment-mediated drug resistance cannot be ignored (11). To increase the proportion of patients potentially cured by long-term TKI therapy molecules, combination strategies are being evaluated currently. However, the development of a combinatorial strategy requires a more detailed investigation of the molecular mechanisms of CML. A better understanding of CML and its underlying molecular mechanisms would increase the accuracy and effectiveness of the effort.

MicroRNAs (miRNAs) are a group of single non-coding RNAs (approximately 22 nucleotides in length). They act as target-specific epigenetic regulators by modulating gene expression through translational repression or mRNA excision (12). Dysregulation of the expression pattern of miRNAs may have many effects, including promoting tumorigenesis (13). MicroRNAs may be implicated in the acquisition of drug resistance in CML as they regulate the expression of not only BCR-ABL1 but also genes associated with the activation of drug transfer proteins or essential signaling pathways (14, 15). Our previous study found that miR-342-5p could target CCND1 to affect imatinib resistance by assessing the differential expression of miRNAs in peripheral blood mononuclear cell (PBMC) from CML patients compared to healthy donors (16). Nonetheless, the roles and functions of many differentially expressed miRNAs (DE-miRNAs) in our studies remain unexplored.

To understand the impact of specifically expressed miRNAs in chronic myeloid leukemia and their target genes, we collected peripheral blood mononuclear cells from patients diagnosed with CML and healthy donors to determine whole miRNA expression by small RNA sequencing and screened out 31 differentially expressed micro RNAs (DE-miRNAs) with high expression. With the utilization of miRNA set enrichment analysis tools, we present here a comprehensive analysis of the relevance of DE-miRNAs to disease and biological function. Furthermore, the literature-based miRNA-target gene database was used to analyze the overall target genes of the DE-miRNAs and to define their associated biological responses. We further integrated DE-miRNA target genes to identify CML miRNA targeted gene signature singscore (CMTGSS) and used gene-set enrichment analysis (GSEA) to analyze the correlation between CMTGSS and Hallmark biological response in PBMC samples from clinical CML patients. Finally, the association of CMTGSS stratification with multiple CML cell lineage gene sets was validated in PBMC samples from CML patients using GSEA. It is hoped that the elucidation of the associated biological functions will narrow the range of options for combination therapeutic strategies and thereby increase the success of the clinical application of the associated inhibitors and treatments.

## Materials and methods

### PBMC clinical sample collection

Informed consent was obtained from each patient and health volunteer for the collection of all samples in accordance with the Helsinki Declaration and institutional guidelines. Ethical approval was obtained from the Institutional Review Board of the Tri-Service General Hospital, and all experimental protocols and methods were performed in accordance with the relevant protocols and regulations. According to the WHO Classification of Tumors of Hematopoietic and Lymphoid Tissues, five samples were collected from newly diagnosed CML patients in chronic phase without any prior treatment, and five normal samples were collected from healthy volunteers after passing the medical examination. Please refer to our previous publications for sample collection, next generation sequencing and processing procedures (16).

### Identification of differentially expressed microRNA

Differentially expressed miRNAs between pairs were analyzed by using the edgeR package in the R software. For each miRNA, significant *p-values* and false discovery rates (FDR) were obtained based on a negative binomial distribution model. The fold change in gene expression was also estimated by the edgeR package. The criteria for DE-miRNA have been set as |log_2_ fold change | >1, log_2_ CPM >4 and FDR < 0.05.

### DE-miRNA-related biological function and target gene associated functional pathway analysis

TAM 2.0 is a web-based tool that uses the published literature on miRNA-related function and pathology as the basis for database building, with family-sets, cluster-sets, disease, function, transcriptional factor (TF) and tissue specificity sets analysis. Regarding the use of the Comparison Wizard function, the 19 up-regulated and 11 down-regulated miRNA lists were entered into the Comparison page, and the list was submitted with the default setting, and the results were filtered with “Leukemia” as the keyword and presented in Bar plot. For Analysis Wizard, enter the up-regulated and down-regulated miRNA list into the Analysis page and submit the list with the default settings. The results were first pre-screened by FDR < 0.25, and the results of Cluster-sets, Cell specificity, and Transcription factor were further filtered by overlapping 2 miRNAs or more; Disease ontology was filtered by the keyword “ Leukemia” was used to filter the results; Function presented only the top 5 results with the smallest FDR. Using R-studio (2022.12.0 Build 353) based on R 4.2.0, the chordDiagram() function in the circlize package is used for miRNA and enriched gene set association plotting (17). Regarding the enriched functional pathways of target genes, 131 up-regulated DE-miRNA target genes and 30 down-regulated DE-miRNA target genes were entered into the Cytoscape (v3.9.1) ClueGo app respectively. The Database is set to Gene Ontology biological processing and is visualized with default parameters. Regarding the miRNA target genes and number of interactions, miRTarBase was set as the analysis engine in mienturnet to analyze the DE-miRNA target genes. The results were filtered by number of interactions ≥ 3, and 131 up-regulated DE-miRNA target genes were obtained (FDR<0.05). The number of down-regulated DE-miRNA target genes was much less than that of up-regulation, so the standard was widened to FDR<0.25 and 30 down-regulated DE-miRNA target genes were obtained. The obtained miRNAs and target genes were visualized by importing the results of mienturnet analysis into the chordDiagram() function in the circlize package.

### Establishment of CML miRNA targeted gene signature singscore using single sample scoring approach

Singscore is a rank-based measure of gene set enrichment in a single sample (18). By scoring both up- and down-regulated gene sets based on the same gene expression ranking, the down-regulated gene set scores are reversed and integrated to obtain a single score. The integrated scores can therefore provide a comprehensive characterization of the transcriptomics of individual samples when both gene expression groups are assessed simultaneously. In this study, miRNAs theoretically inhibit the mRNA expression or translation of target genes, so that the expression of target genes with down-regulated miRNAs increases, while the expression of target genes with up-regulated miRNAs decreases. To reasonably integrate the difference between the two into a single score, we set up- and down-regulated gene sets as up- and down-regulated miRNA target genes in the singscore package (v1.18.0) respectively. The lower CMTGSS obtained represents a decrease in the expression of the up-regulated miRNA target gene cluster and an increase in the expression of the down-regulated miRNA target gene cluster. The CMTGSS of all samples in the individual databases were standardized by Z-score, with a cut-off of 0. Samples greater than or equal to 0 were considered as CMTGSS high and those less than 0 were considered as CMTGSS low. For differential expression analysis based on CMTGSS stratification, the Wilcoxon signed-rank test was used, considering the large sample size of individual databases (19). Differentially expressed genes and their fold changes were sorted in descending order as genelist and submitted to the clusterprofiler for subsequent analysis. Please refer to the **Supplementary Table** for the components of the CMTGSS that contribute to the up- and down-regulated genes.

### GEO database access and immune cell infiltration score assessment

The datasets supporting the results of this paper are available in the NCBI Gene Expression Omnibus and can be accessed through the GEO series registries GSE144119 and GSE72316. GSE144119 data have been downloaded and converted from counts to transcripts per million (TPM) for subsequent analysis. To quantify the abundance of stromal cells and immune cells in the PBMC of CML patients, the xCell package in R was used to estimate the scores of 64 infiltrating cell subtypes from the normalized RNA sequencing data. After calculating cell scores for each sample, signal-to-noise and similarity matrix test based on CMTGSS stratification were performed using Morpheus web tool (https://software.broadinstitute.org/morpheus/).

### Gene set enrichment analysis

GSEA analysis was performed according to CMTGSS stratification, using the GSEA() function of the ClusterProfiler package (v4.6.0) with default settings. The corresponding NES of each gene set is plotted by ggbarplot(). Concerning ridgeplot, it is drawn with the ridgeplot() function of the ClusterProfiler package. The gene sets used in this study include CML cell division by Graham et al. (20), CML gene set by Diaz et al. (21), HSC properties by Eppert et al. (22), LSC profiling by Gal et al. (23), Zheng et al. published Cord blood hematopoietic cell lineage (24), and bone marrow derived cell population by Hay et al. (25). Hallmark gene-set was obtained from the MsigDB database (h.all.v2022.1.Hs.symbols.gmt). Apoptosis and cell cycle associated gene-set was downloaded from the BioPlanet database (26).

## Results

### Identifying the DE-miRNAs between CML patients and healthy donors

**Figure 1:**
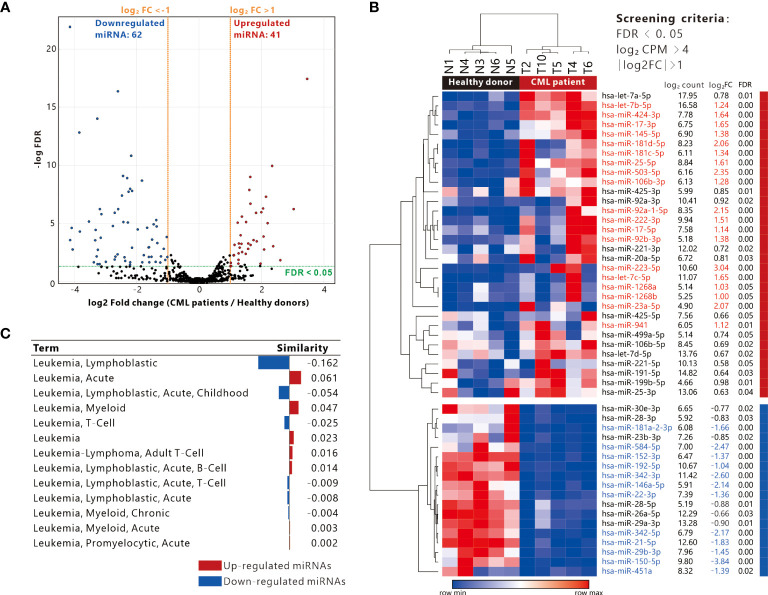
Definition of differentially expressed miRNAs and association with leukemia. **(A)** Volcano plot showing the distribution of up-regulated and down-regulated miRNAs (screening criteria: |Log_2_ fold change (FC)| >1, FDR < 0.05). **(B)** Heatmap presenting the expression of differentially expressed miRNAs in 5 CML patients and 5 healthy donor PBMC samples (filtering criteria: Log_2_ CPM > 4, FDR < 0.05), red and blue text indicate miRNAs with log_2_ FC > 1 and < -1 respectively. **(C)** Analysis of differentially expressed genes and disease similarity using TAM2.0.

In our previous study, we collected total RNA from PBMC samples from 5 patients with CP-CML and healthy donors for miRNA sequencing, and 2,590 miRNAs have been determined (16). Differentially expressed miRNAs (DE-miRNA_ were analyzed by EdgeR. We further identified 103 DE-miRNAs (FDR < 0.05), of which 62 miRNAs were down-regulated and 41 miRNAs were up-regulated (**Figure 1A**). Considering the miRNA expression abundance, we conducted another screening with the criterion of log_2_ CPM > 4 and obtained 32 up-regulated miRNAs and 18 down-regulated miRNAs (**Figure 1B**). There were 30 miRNAs with absolute log_2_FC > 1, 19 up-regulated DE-miRNAs (in red) as well as 11 down-regulated DE-miRNAs (in blue). Pathological association analysis of DE-miRNAs using the publicly available bioinformatics tool TAM 2.0, with “leukemia” as the keyword for screening, showed that DE-miRNAs were associated with a variety of leukemias. The upregulated DE-miRNAs were more associated with “Acute or Myeloid leukemia”, while the downregulated DE-miRNAs were more similar to “Lymphoblastic leukemia” (**Figure 1C**).

### Upregulated DE-miRNAs and their target genes are associated with apoptosis

**Figure 2:**
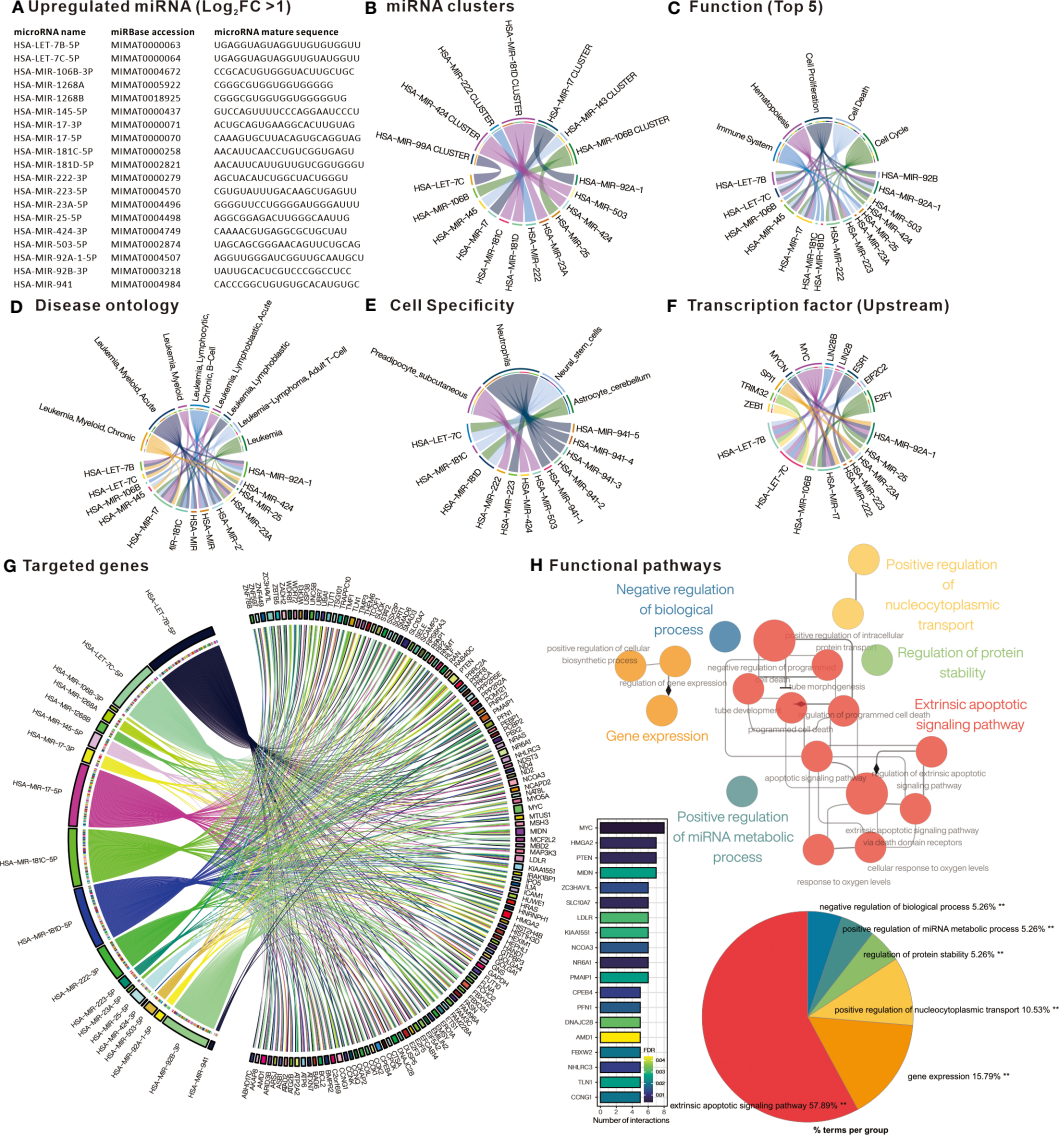
Comprehensive analysis of upregulated differentially expressed miRNAs. **(A)** List of miRNAs defined as significantly upregulated in PBMC of CML patients. Chord plots present **(B)** miRNAs belonging to cluster family; **(C)** related functions; **(D)** Disease ontology; **(E)** Cell specificity; and **(F)** upstream regulatory transcription factors, as defined by TAM2.0. **(G)** Chord diagram presenting miRNA target genes defined by Mienturnet based on mirTarBase, bar chart presenting genes interfered by more than 5 miRNAs. **(H)** Significantly correlated Gene Ontology biological processing gene sets of up-regulated miRNA target genes analyzed by Cytoscape ClueGO.

In order to investigate the potential impact of DE-miRNA aberrant expression, the trend of miRNA in CML patient PBMC was divided into up-regulation and down-regulation. The upregulated miRNAs were analyzed by TAM2.0 (**Figure 2A**), showing that the most upregulated miRNAs belonged to the HAS-miR-181D cluster (**Figure 2B**). For function, the upregulated miRNAs were enriched in cell proliferation, cell cycle and cell death (**Figure 2C**). Disease ontology, pre-screened by the keyword “Leukemia”, shows that the most upregulated DE-miRNAs are associated with “Leukemia, Myeloid, Acute” (**Figure 2D**). In terms of cell specificity, most upregulated DE-miRNAs were mostly associated with “Neutrophils” (**Figure 2E**). Upstream transcription factors such as MYC, E2F1, SPI1 and ESR1 dominate the upregulated expression of most miRNAs (**Figure 2F**). We used Mienturnet to analyze the potential target genes of upregulated DE-miRNAs and showed that genes including MYC, HMGA2, PTEN and MIDN were the majority of upregulated miRNA targets (**Figure 2G**). Cytoscape ClueGO analysis of the functional pathways associated with the target genes of the upregulated DE-miRNA showed that the Extrinsic apoptotic signaling pathway was the most relevant (**Figure 2H**). This suggests that DE-miRNAs upregulated in PBMC of CML patients may affect drug resistance in CML cells by interfering with the expression of apoptosis-related genes.

### Down-regulated DE-miRNAs and their target genes are associated with cell cycle regulation

**Figure 3:**
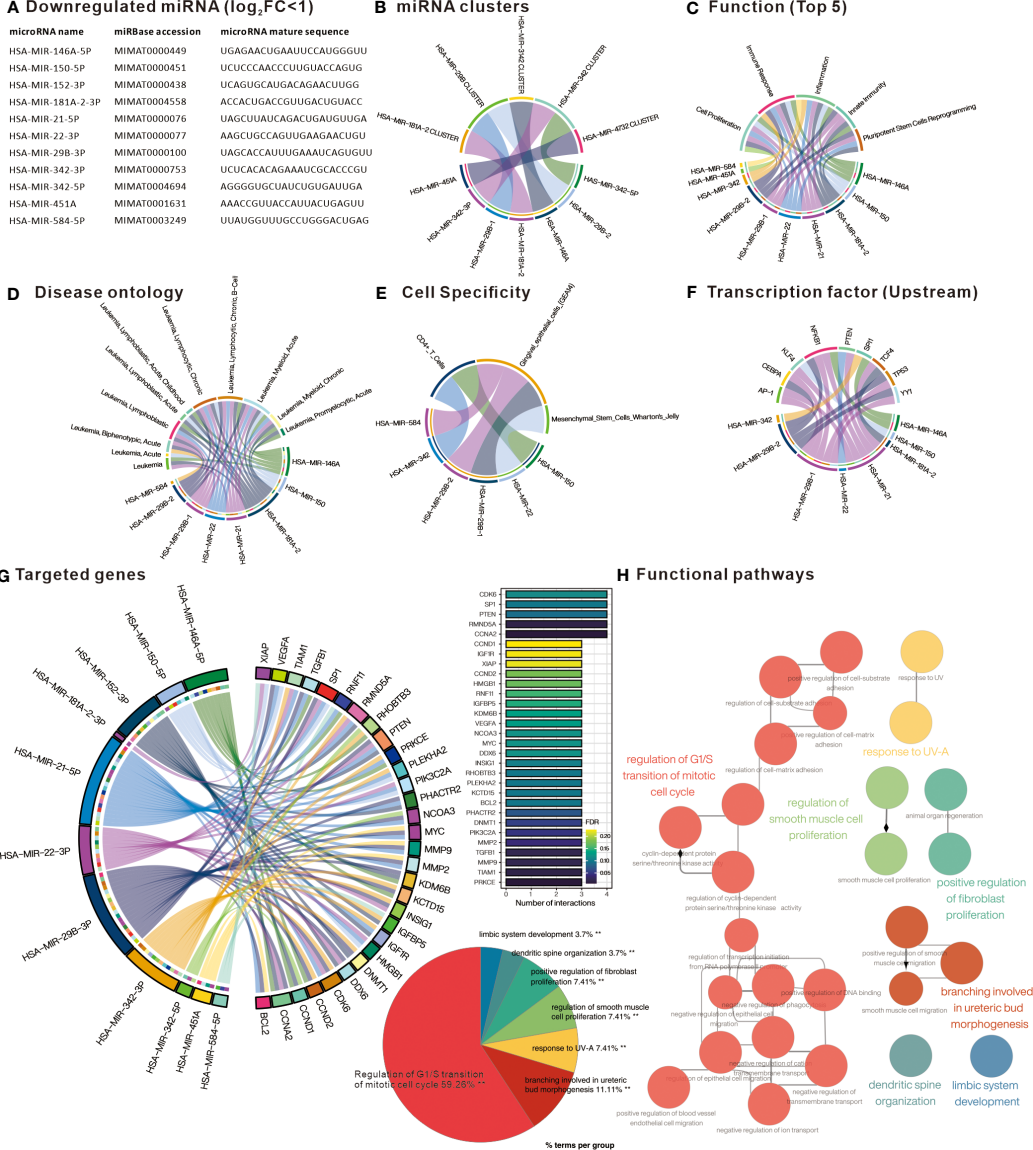
Comprehensive analysis of downregulated differentially expressed miRNAs. **(A)** List of miRNAs defined as significantly downregulated in PBMC of CML patients. Chord plots present **(B)** miRNAs belonging to cluster family; **(C)** related functions; **(D)** Disease ontology; **(E)** Cell specificity; and **(F)** upstream regulatory transcription factors, as defined by TAM2.0. **(G)** Chord diagram presenting miRNA target genes defined by Mienturnet based on mirTarBase, bar chart presenting genes interfered by more than 3 miRNAs. **(H)** Significantly correlated Gene Ontology biological processing gene sets of down-regulated miRNA target genes analyzed by Cytoscape ClueGO.

Similarly, analysis of the down-regulated DE-miRNAs (**Figure 3A**) into TAM2.0 showed that most of the miRNAs belonged to the HSA-miR-29B and HSA-miR-342 clusters (**Figure 3B**). In terms of function, down-regulated DE-miRNAs were enriched in Cell proliferation, Immune response, and Inflammation (**Figure 3C**). Disease ontology shows that the most upregulated DE-miRNAs are associated with “Leukemia, Myeloid, Acute”, similar with upregulated DE-miRNAs (**Figure 3D**). Regarding cell specificity, the more relevant blood cells are CD4^+^ T cells (**Figure 3E**). As for upstream transcription factors, NF-kB is the regulator of most down-regulated DE-miRNAs (**Figure 3F**). Mienturnet analysis of down-regulated DE-miRNA potential target genes showed that genes including CDK6, SP1, PTEN, RMND5A, CCNA2 were most affected (**Figure 3G**). Analysis of the biological functions of the target genes by Cytoscape ClueGO showed that the Regulation of G1/S transition of mitotic cell cycle was most associated with the genes targeted by down-regulated DE-miRNAs (**Figure 3H**). This implies that the down-regulated miRNAs in PBMC of CML patients may have the function of suppressing the expression of cell cycle-related genes and may indirectly promote the proliferation of CML cells after aberrant down-regulation.

### Assessment of individual miRNA expression in PBMC of CML patients in relation to clinical disease progression

**Figure 4:**
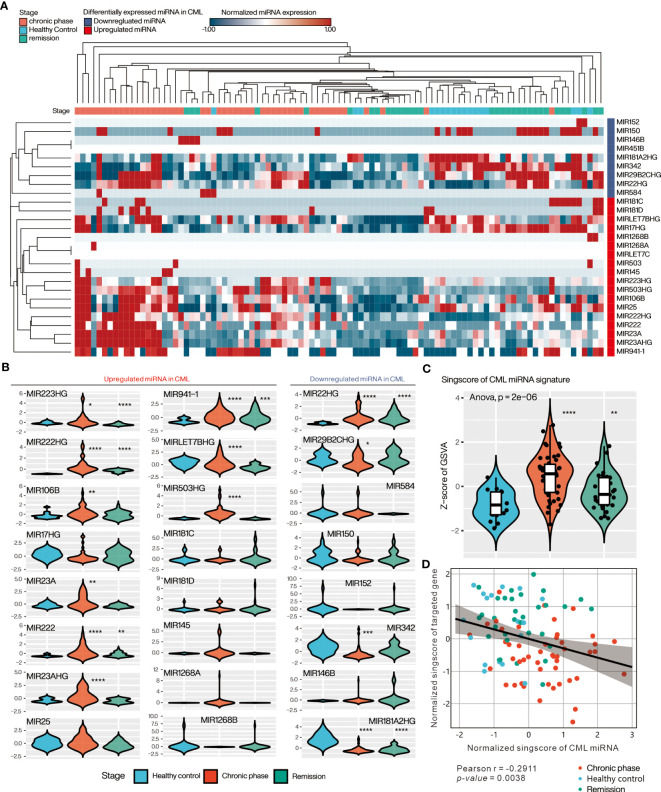
Evaluation of DE-miRNA expression in PBMC of clinical CML patients in the GSE144119 database. **(A)** Heatmap presents the overall expression of DE-miRNAs in CML patient samples, ordered by Hierarchical clustering with Euclidean distance. **(B)** Violin plot showing the relative expression distribution of each miRNA in the three clinical stages of CML. The expression of each miRNA was pre-normalized by z-score method. **(C)** Violin plot presenting the relative expression distribution of singscore of CML miRNA signature in the three CML stages. **(D)** Correlation analysis of singscore of CML miRNA signature and singscore of CML miRNA targeted gene-set. One-way ANOVA was used to assess the statistical significance of between-group differences. Students’ t-test was conducted to assess the significance of the difference between each stage and healthy control. *: p<0.05, **: p<0.01, ***: p<0.001, ****: p<0.0001.

We further verified the expression of differential miRNAs in 97 samples from CML patients at diagnosis or remission and from healthy donors using the GSE144119 database. Heatmap with Hierarchical clustering was used to visualize the distribution of differential miRNAs in the samples, showing that most of the up-regulated DE-miRNAs were highly expressed in the chronic phase, while the down-regulated DE-miRNAs were mainly in healthy control or remission (**Figure 4A**). Comparing individual miRNAs, most of the up-regulated DE-miRNAs were significantly increased in CML samples compared to healthy donors (miR-223, miR-222, miR-106B, miR-23A, miR-LET-7B, miR-106B, miR-503), and decreased after treatment reached remission. In contrast, down-regulated DE-miRNAs were significantly reduced in CML samples relative to healthy donors (miR-2982C, miR-342, miR-181A2HG), and increased after treatment to achieve response (**Figure 4B**). These results suggest that aberrant expression of DE-miRNA is associated with the progression of CML and recovers to a state close to that of a healthy donor after treatment. Using the singscore method to integrate upregulated and downregulated miRNAs’ expression as a single value, we showed that the CML miRNA singscore increased significantly in the chronic phase and decreased in patients who reached remission, suggesting that the overall miRNA expression we found reflects the disease progression of clinical CML patients (**Figure 4C**). We further tested whether the overall miRNA target gene expression was inversely correlated with the miRNA singscore. We used the singscore approach to integrate the DE-miRNA targeted gene from **Figures 2G** and **3G** and showed that the CML miRNA singscore was negatively correlated with the CML miRNA targeted gene signature singscore (CMTGSS) (Pearson correlation coefficient r = -0.2911, *p-value* = 0.0038), suggesting that miRNA expression is negatively correlated with its target gene in clinical samples (**Figure 4D**).

### Low CMTGSS is associated with disease progression, cell proliferation, and immunosuppression

**Figure 5:**
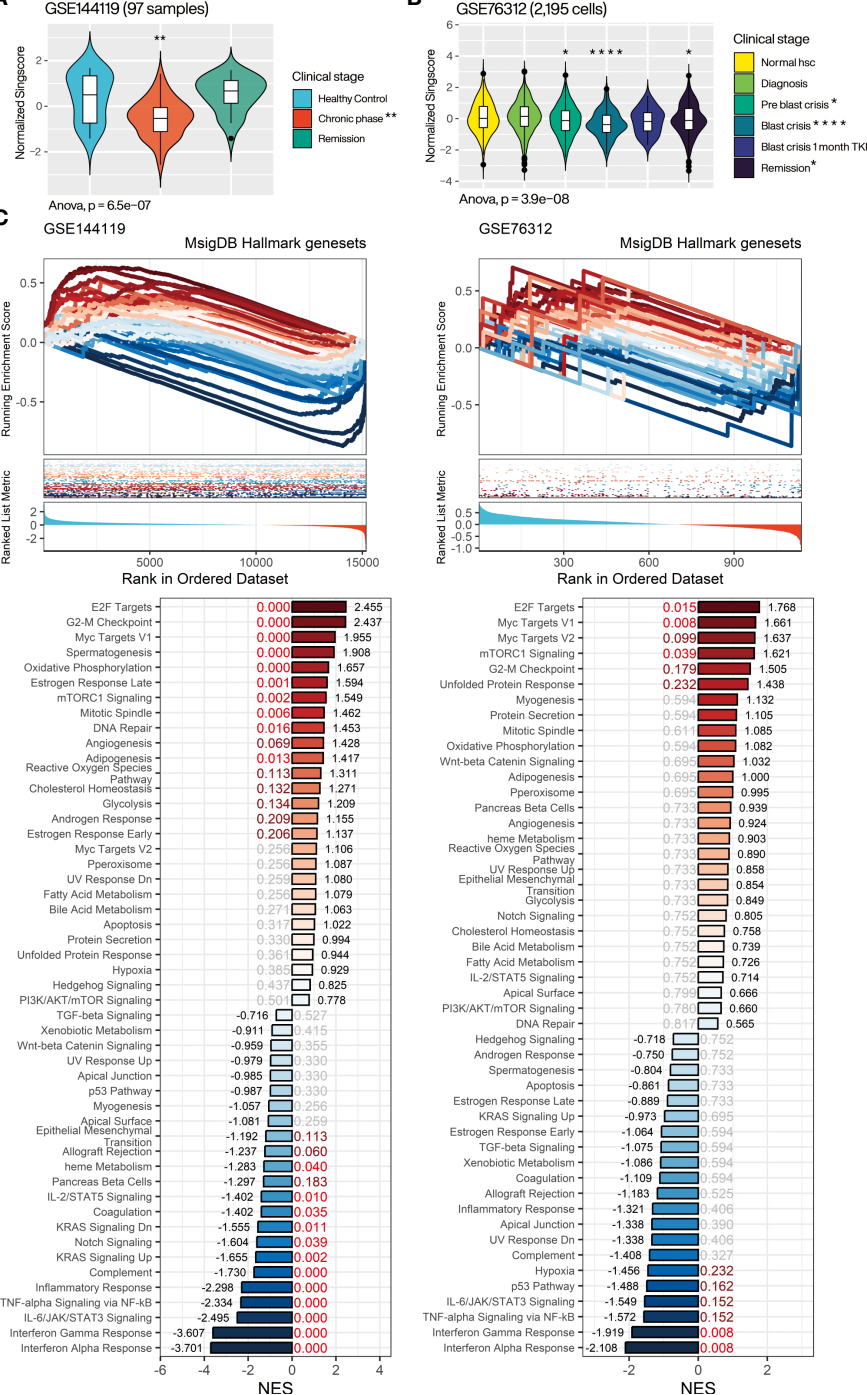
Assessment of the relevance of CMTGSS to the clinical stage of CML and 50 Hallmark gene sets. Violin plot presents the relative expression distribution of CMTGSS in **(A)** GSE144119 for CML patients from clinical stages of healthy control, chronic phase, and remission and **(B)** GSE76312 for CML patients from normal HSC, diagnosis, pre blast crisis, blast crisis, blast crisis with 1 month TKI treatment, and remission. **(C)** GSEA presents CMTGSS stratification and enrichment of 50 Hallmark gene-set, CMTGSS is divided into low CMTGSS and high CMTGSS with z-score = 0. NES is displayed at the end of the bar plot, FDR is highlighted in the center, and the red text represents FDR < 0.25. One-way ANOVA was used to assess the statistical significance of betweengroup differences. Students’ t-test was conducted to assess the significance of the difference between each stage and healthy control. *: p<0.05, **: p<0.01, ***: p<0.001, ****: p<0.0001.

Aiming to resolve the association of CML miRNA targeted gene with clinical progression, we evaluated the overall expression of CMTGSS in different disease progressions in the GSE144119 and GSE76312 databases (**Figure 5A**). GSE144119 has 97 PBMC samples from clinical CML patients and GSE76312 contains 2,195 CD34^+^ cells from PBMC of patients with differing clinical stages of CML. In GSE144119, CMTGSS was significantly lower in the chronic phase, which was consistent with the elevated expression of the overall CML miRNA singscore (**Figure 4C**). Singscore evaluation of 2,195 cells in GSE76312 showed a significant decrease in CMTGSS in cells from both the Pre blast crisis and Blast crisis clinical phases. This was followed by a rebound at 1 month of TKI treatment and at the Remission clinical stage, suggesting a potential association between BCR-ABL activity and miRNA expression (**Figure 5B**). To assess the association of CMTGSS with various cancer biological functions, we used the Hallmark gene-set integrated by the MSigDB team to analyze the 50 biological response enrichment of all samples in GSE144119 and GSE76312 by GSEA (**Figure 5C**). We standardized the CMTGSS to a Z score for the grouping (Z score > 0: CMTGSS high, Z score ≤ 0: CMTGSS low). The results showed that E2F TARGETS, G2M CHECKPOINT, MYC TARGETS V1 and MTORC1 SIGNALING were significantly positively enriched in both databases in CML patients with low CMTGSS, while IL6 JAK STAT3 SIGNALING, TNFA SIGNALING VIA NFKB, INTERFERON GAMMA RESPONSE and INTERFERON ALPHA RESPONSE were significantly negatively enriched, suggesting that low CMTGSS is associated with enhanced CML cell proliferation and suppression of immune responses.

Several cell cycle-related gene sets were significantly positively associated with CML patients with low CMTGSS populations, echoing our previous results, but apoptosis did not reach significant enrichment in either database, suggesting that the association with apoptosis inhibition may be restricted to specific mechanisms. We further analyzed the association of CMTGSS stratification with apoptosis and cell cycle regulation in GSE144119 using the more inclusive BioPlanet gene set (26). The results showed that low CMTGSS patients were only negatively enriched with the set of genes associated with apoptosis induced by immune cells and remained positively enriched with a variety of cell cycle gene sets, particularly G1 to S cell cycle control (**Supplementary Figure**).

### Low CMTGSS is associated with positive enrichment of erythroid and granulocyte macrophage progenitor gene sets

**Figure 6:**
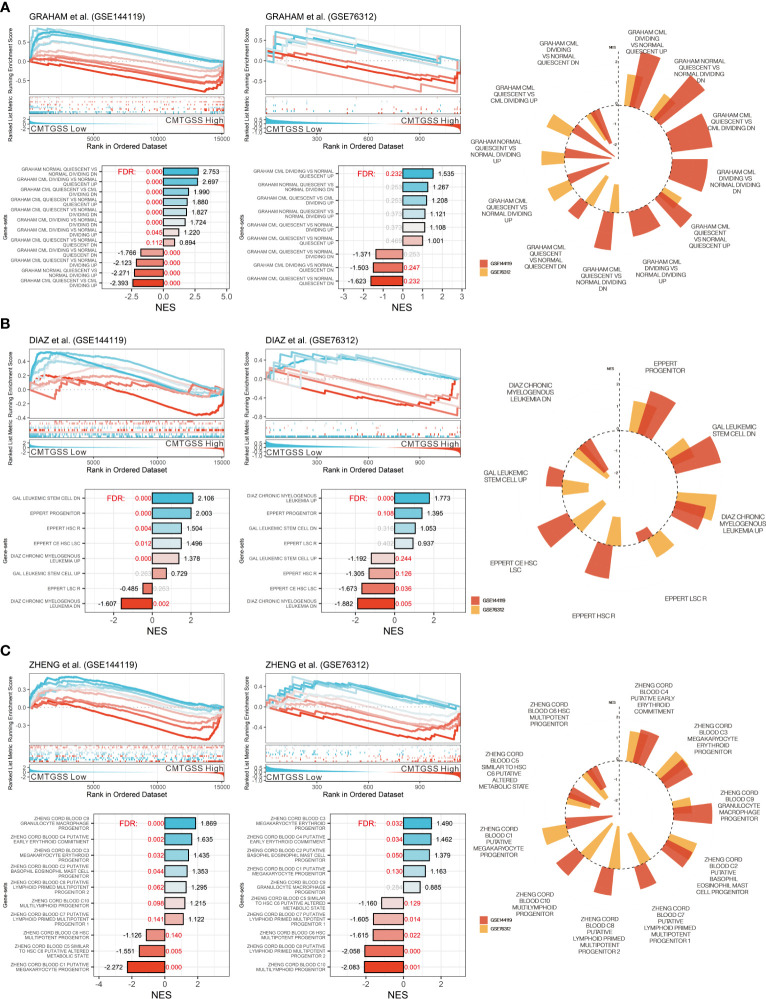
Evaluation of CMTGSS stratification and enrichment of multiple CML-associated gene-sets. GSEA assessed the enrichment of low CMTGSS with **(A)** the CML proliferation gene set from Graham et al. and **(B)** multiple CML cell property-related gene sets, and **(C)** 10 cord blood-derived blood cell types from Zheng et al. in GSE144119 and GSE76312. Normalized enrichment scores (NES) are displayed at the end of the bar plot, The value of false discovery rate (FDR) is highlighted in the center, and the red text represents FDR < 0.25. Radar plots present gene sets with the same enrichment tendency in both databases.

To better delineate the relationship between CMTGSS and the various blood cells involved in the development of CML, we first performed a GSEA (**Figure 6A**) using the CML division-associated gene set from Graham et al. CML dividing *vs.* normal quiescent up was significantly enriched in the low CMTGSS group (FDR < 0.001, NES = 2.753 in GSE144119; FDR = 0.232, NES = 1.535 in GSE76312), suggesting that PBMC in patients with low CMTGSS is associated with higher CML division. Analysis with multiple hematopoietic stem cell-associated gene sets showed a significant positive enrichment of the low CMTGSS group with CML up (FDR < 0.001, NES =1.378 in GSE144119; FDR < 0.001, NES = 1.773 in GSE76312) as well as progenitor (FDR < 0.001, NES = 2.003 in GSE144119; FDR = 0.106, NES = 1.395 in GSE76312) (**Figure 6B**). In view of the positive progenitor enrichment, it is suggested that the degree of CMTGSS may reflect the progenitor differentiation tendency of a specific group of blood cells. GSEA analysis of the ten blood cell gene sets provided by Zheng et al. showed that C4 putative early erythroid commitment, C3 megakaryocyte erythroid progenitor, C9 Granulocyte macrophage progenitor, and C2 putative basophil eosinophil mast cell progenitor were positively enriched in the low CMTGSS group, suggesting that aberrant expression of miRNAs may affect the distribution of related progenitors in the blood by regulating their target genes (**Figure 6C**).

### Assessment of the association of CMTGSS expression with bone marrow differentiating cell population

**Figure 7:**
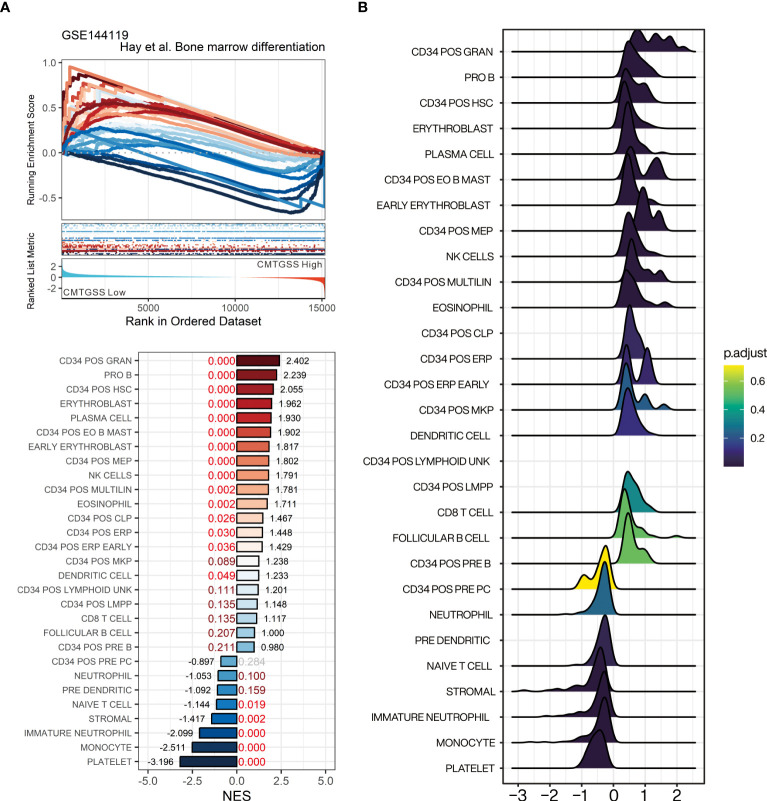
Evaluation of gene-set enrichment of low CMTGSS stratification in bone marrow-derived cell lineage by GSEA. **(A)** GSEA presents the enrichment of 23 blood cell lineages related to bone marrow differentiation. NES is displayed at the end of the bar plot, FDR is highlighted in the center, and the dark red text represents FDR < 0.25, red text stands for FDR < 0.05. **(B)** Ridge plot shows the fold change distribution of genes in each bone marrow gene set. Please refer to the section “Abbreviation” for the cell lineage represented by the individual gene sets.

For the purpose of assessing the linkage between miRNA targeted gene and blood cell distribution, we performed GSEA using the bone marrow cell lineage composition gene set published by Hay et al. to assess the association between the enrichment of blood cells and the CMTGSS stratification (25). The results showed that cell populations of CD34^+^ granulocyte (NES = 2.402, FDR < 0.001), pro B cell (NES = 2.239, FDR <0.001), CD34^+^ HSC (NES = 2.055, FDR < 0.001), and erythroblast (NES = 1.962, FDR < 0.001) were positively enriched in CML patients with low CMTGSS. We also found that naïve T cell (NES = -1.144, FDR = 0.019), immature neutrophil (NES = -2.099, FDR < 0.001), monocyte (NES = -2.511, FDR < 0.001), and platelet (NES = -3.196, FDR < 0.001) were negatively enriched in patients with low CMTGSS (**Figure 7A**). Ridgeplot showed the overall gene distribution of each bone marrow differentiated cell population (**Figure 7B**), suggesting that the expression of CML miRNAs may be potentially associated with the distribution of the above cell populations.

### Evaluation of the association of the CMTGSS stratification with various immune components using a simulated immune infiltration strategy

**Figure 8:**
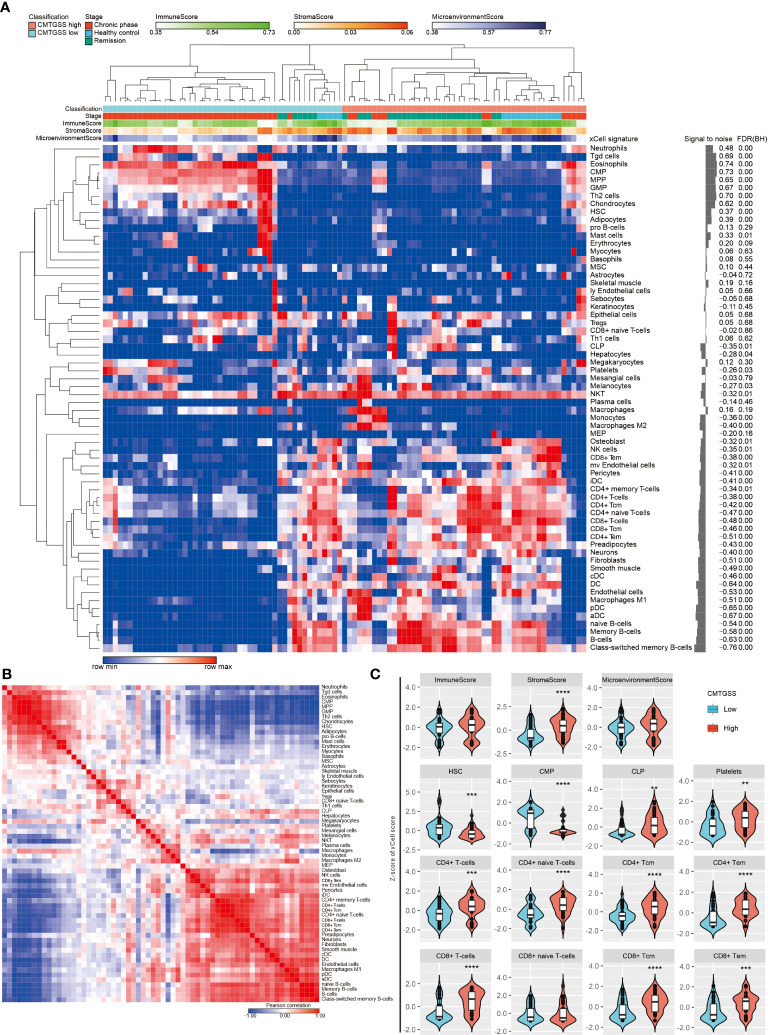
Assessment of the correlation between CMTGSS and multiple cell infiltration levels by xCell method. **(A)** Heatmap presentation of the distribution of CMTGSS classification and blood cellular heterogeneity landscape of CML patients in the GSE144119 database, sorted by hierarchical clustering (One minus Pearson correlation). **(B)** Similarity matrix of xCell immune cell type gene features of CML patients in the GSE144119 database. The 64 immune cell type gene features were paired with each other. Similarity was calculated by the Pearson correlation test to calculate the degree of overlap of each pair of xCell scores. Colors represent Pearson correlation coefficient, red indicates high similarity, blue indicates negative similarity. White indicates no correlation. **(C)** Violin plots demonstrate differences in Z-score across simulated immune and stromal cell infiltration of CMTGSS classification; Students’ t-test was used to assess the significance of the differences; *: *p* < 0.05, **: *p* < 0.01, ***: *p* < 0.001, ****: *p* < 0.0001. Please refer to the section “Abbreviation” for the cell lineage represented by the individual cell types.

Given the negative enrichment of naive T cells in patients with low CMTGSS, we were curious whether the overall expression of CML miRNA targeted gene was associated with the infiltration of other immune cells. We analyzed the GSE144119 database using the xCell method, and the CMTGSS-stratified heatmap showed that the 64 immune cell scores were broadly divided into two regions (**Figure 8A**). According to the similarity matrix, the upper left region includes CMP lineage innate immune cells such as neutrophil and eosinophils, which are significantly higher in the low CMTGSS, while the lower right region is dominated by CLP lineage adaptive immune cells, including CD4, CD8 and B cells, which are significantly higher in the low CMTGSS (**Figure 8B**). The xCell score was then used to plot a violin plot to assess the distribution and differences between groups, showing that the stromal score was significantly lower in the Low CMTGSS, while there was no significant difference in the Immune score or microenvironment score (**Figure 8C**). With regard to blood cell differentiation lineage, HSC was higher in low CMTGSS group, while CMP and CLP scores were significantly diverged, that is, low CMTGSS group had higher CMP and lower CLP, suggesting that miRNA-targeted gene expression was associated with CMP enrichment. Platelets belonging to the CMP lineage had lower scores in the Low CMTGSS group, indicating that miRNA target genes may influence platelet differentiation and maturation. In contrast, the CD4^+^ and CD8^+^ T cell families belonging to the CLP lineage were significantly lower in the low CMTGSS, implying that the specific expression of miRNAs in CML may have an inhibitory effect on CLP differentiation.

## Discussions

The individual miRNA variations and effects on PBMC in CML patients have been investigated in detail in many studies, as well as the abnormal reduction of miR-342-5p in PMBC of CML patients and its effect on imatinib resistance, which we previously investigated. Several integrative studies have also demonstrated the potential of miRNAs to predict the progression of CML (27, 28). However, there is still a lack of assessment of the hematological microenvironment and prognosis of CML patients in terms of interference with their target genes by aberrantly expressed miRNAs. In this study, 19 up-regulated miRNAs and 11 down-regulated miRNAs were defined in CML patients using previous miRNA sequencing results collected from PBMC of CML patients and healthy donors. As far as the published literature is concerned, the effect of upregulated miRNAs on CML is not always consistent. For example, Let-7, miR-23A, and miR-223 have been reported to act as tumor suppressors in CML (29–31). miR-145 and miR-181 promote apoptosis of leukemia stem cells through regulation of ABCE1 and MCL-1 respectively (32, 33), and miR-424 inhibits BCR-ABL activity (34). Comparatively, the miR-126 is associated with leukemia stem cell maintenance (35), miR-17 promotes leukemia proliferation by targeting p21 (36), over-expression of miR-106 and miR-222 will promote CML proliferation (37), and miR-92a-1-5p inhibits TKI-induced necroptosis by targeting MLKL (38). However, in general, the genes targeted by up-regulated miRNAs are mostly associated with Extrinsic apoptotic signaling pathways, suggesting that when these genes are interfered by up-regulated DE-miRNAs, they could potentially affect the apoptosis of cells. In terms of downregulated miRNAs, decreased miR-146A and miR-150 have been reported to be associated with CML (39, 40), with the former possibly being associated with regulation of NF-κB-driven inflammation and leukemia progression (41). miR-152-3p promotes CML development by inhibiting p27 (42), miR-342-5p inhibits proliferation caused by BCR-ABL and resistance to imatinib by targeting CCND1 (16), and miR-584 has been reported to have a possible role as a tumor suppressor in lung cancer (43). Most of the genes targeted by down-regulated DE-miRNA are associated with the regulation of G1/S transition of mitotic cell cycle, and when these genes are not inhibited by down-regulated miRNAs, they may rise abnormally and promote cell cycle and proliferation.

Confirmation of the individual and overall miRNA expression profiles by the GSE144119 database showed an increasing trend in miRNA expression during the chronic phase, in contrast to its target genes, and a decrease to a similar level to healthy control when the patient was in remission (**Figure 4**). In line with this observation, a significant decrease in target gene singscore was detected in cells within pre-blast crisis and blast crisis phases in the single cell database of GSE76312. Furthermore, the CMTGSS of blast crisis CML patients increased after one month of TKI administration, suggesting that BCR-ABL1 activity may be responsible for the aberrant expression of miRNAs and that TKI administration may correct the expression of miRNAs. Even TKI intervention may reverse the aberrant miRNA expression, the proliferative and anti-apoptotic effects of aberrantly expressed miRNAs in the hematological microenvironment may still deliver CML cells with resistance to TKI before significant remission is achieved. This suggests that the synergistic use of TKI and anti-apoptotic inhibitors may be effective in relieving the microenvironmental interference caused by miRNAs, with a number of encouraging reports of success (44–47).

Analysis of the Hallmark gene-set showed that the majority of positively enriched gene sets were associated with cell proliferation and revealed a potential response to immunosuppression. Further analysis of the association of CMTGSS expression with various blood cell types from the clinical database suggests that the enrichment of progenitor may be a relevant effect of these DE-miRNAs and may explain the occurrence of granulocyte macrophage progenitor and megakaryocyte erythroid progenitor (48, 49). Validation of the bone marrow-derived cell gene set published by Hay et al. indicates that the overall low expression of miRNA target genes is associated with granulocyte macrophage progenitor, and also suggests a potential association with platelet dysfunction (2). In terms of the association of miRNAs with cells in the blood lineage, aberrantly expressed miRNAs originating from CML cells may further affect other CML or immune cells through the exosome (50, 51). For example, as one of the most upregulated DE-miRNA targets, altered expression of myc may affect the expansion of pro-B cells (52) or the differentiation of HSC or CML to erythroid cells (53–55). It has also been shown that miRNA containing exosomes may affect T cell function and distribution (56).

Studies have evaluated the use of miRNAs in the blood system of CML patients as biomarkers of disease prognosis. Litwińska et al. reviewed recent studies on the important role of miRNAs in the pathogenesis of CML and their relevance as biomarkers for diagnosis, monitoring disease progression and therapeutic response (14). Nevertheless, most of the studies only focus on the differential expression of one to a few miRNAs. The strategy of predicting the occurrence or prognosis of an individual’s disease through bioinformatics with integrative analysis is increasingly employed (57). Zhong et al. combined machine learning with multiple CML databases to screen for four CML diagnostic genes, demonstrating high predictive power and immunosuppressive correlation in a clinical cohort (58). Hue et al. evaluated the differential miRNA expression by small B-cell lymphoma formalin-fixed, paraffin-embedded tissue samples and revealed the correctness of 14 miRNAs for predicting different types of lymphoma (59). Ruiz et al. analyzed the miRNome of the LSC-enriched CD34^+^CD38^-^CD26^+^ fraction in CML-CP patients and found a more than 9-fold increase in miR-196a-5p in the CD26^+^ (BCR-ABL1^+^) versus CD26^-^ (BCR-ABL1^-^) CD34^+^CD38^-^ fraction at diagnosis (60). In this study, a series of analyses based on highly and differentially expressed miRNA populations were performed and validated using samples from clinical databases to obtain an overall DE-miRNA potential association with biological response. For clinical applications, the design of multiple miRNA detection platforms will allow multiple miRNA expression measurements in PBMC cells isolated from the blood of CML patients. If the assessment shows an abnormal increase or decrease in most miRNAs, it may be possible to consider synergistic therapy with Venetoclax and TKIs or to assess the possibility of immunosuppression to increase the success of treatment.

There are several limitations to our study. First, we only collected PBMC from 5 donors each with CML and healthy donor for miRNA sequencing, which may not be a large sample size. However, we selected targets with higher expression and greater fold changes as targets for analysis to increase confidence, and in a previous study we have performed qPCR on some of the miRNAs in additional PBMC samples from 13 healthy donors and 20 CML patients to confirm the existence of differences (16). In terms of validation of the biological function of DE-miRNAs, it is undeniable that integrative analysis of transcriptomics usually lacks solid validation. In contrast, confirming the role of a single miRNA requires repeated validation of multiple aspects to be convincing, as in our previous study of miR-342-5p in CML. If multiple miRNA expressions were to be validated for their effects on biological responses, it would not only be difficult to present a large amount of analytical data, but would also obscure the focus of our goal to assess the association between the overall expression of DE-miRNAs and biological responses. Further, if multiple miRNAs are expressed simultaneously in CML cell lines, in addition to the difficulties in validation, it may be challenging to realistically represent similar responses to miRNA expression in human PBMC using only CML cell lines as a platform. Integrative transcriptional analysis can be used to assess the biofunctional relevance of clinical samples that are closest to the real state, directly presenting the effects of differential gene expression on cancer cells and the surrounding environment, allowing clinicians and researchers to design further studies based on the reported relevance to elucidate the true cause of the disease.

Collectively, this study defined differentially expressed miRNAs by miRNA sequencing from clinical samples and comprehensively analyzed the biological functions of the differential miRNAs in association with the target genes. The analysis of the enrichment of specific myeloid differentiated cells and immune cells also suggests the magnitude and potential targets of differentially expressed miRNAs in the clinical setting. It helps us to make links between the different results obtained from the multi-faceted studies to provide more potential research directions.

## Data availability statement

Publicly available datasets were analyzed in this study. This data can be found here: GSE76312 (https://www.ncbi.nlm.nih.gov/geo/query/acc.cgi?acc=GSE76312); GSE144119 (https://www.ncbi.nlm.nih.gov/geo/query/acc.cgi?acc=GSE144119).

## Ethics statement

The studies involving human participants were reviewed and approved by the Institutional Review Board of Tri-Service General Hospital, Taipei, Taiwan (IRB number: 1-105-05-052). The patients/participants provided their written informed consent to participate in this study.

## Author contributions

Y-YW and S-CW designed the research. X-JL and H-FL performed data analysis. Y-YW, S-CW, Y-GC, P-HC and S-WL acquired clinical samples and parameters from CML patients and healthy donors. Y-LC and C-LH supervised the project and provided funding. The paper was written and reviewed by Y-LC and C-LH. All authors contributed to the article and approved the submitted version.
