# Supplementary figures and images for: Profiling of miRNAs and their interfering targets in peripheral blood mononuclear cells from patients with chronic myeloid leukaemia
Source: Front Oncol. 2023 Jul 5;13:1173970. doi: 10.3389/fonc.2023.1173970 (PMC10356106; doi:10.3389/fonc.2023.1173970)

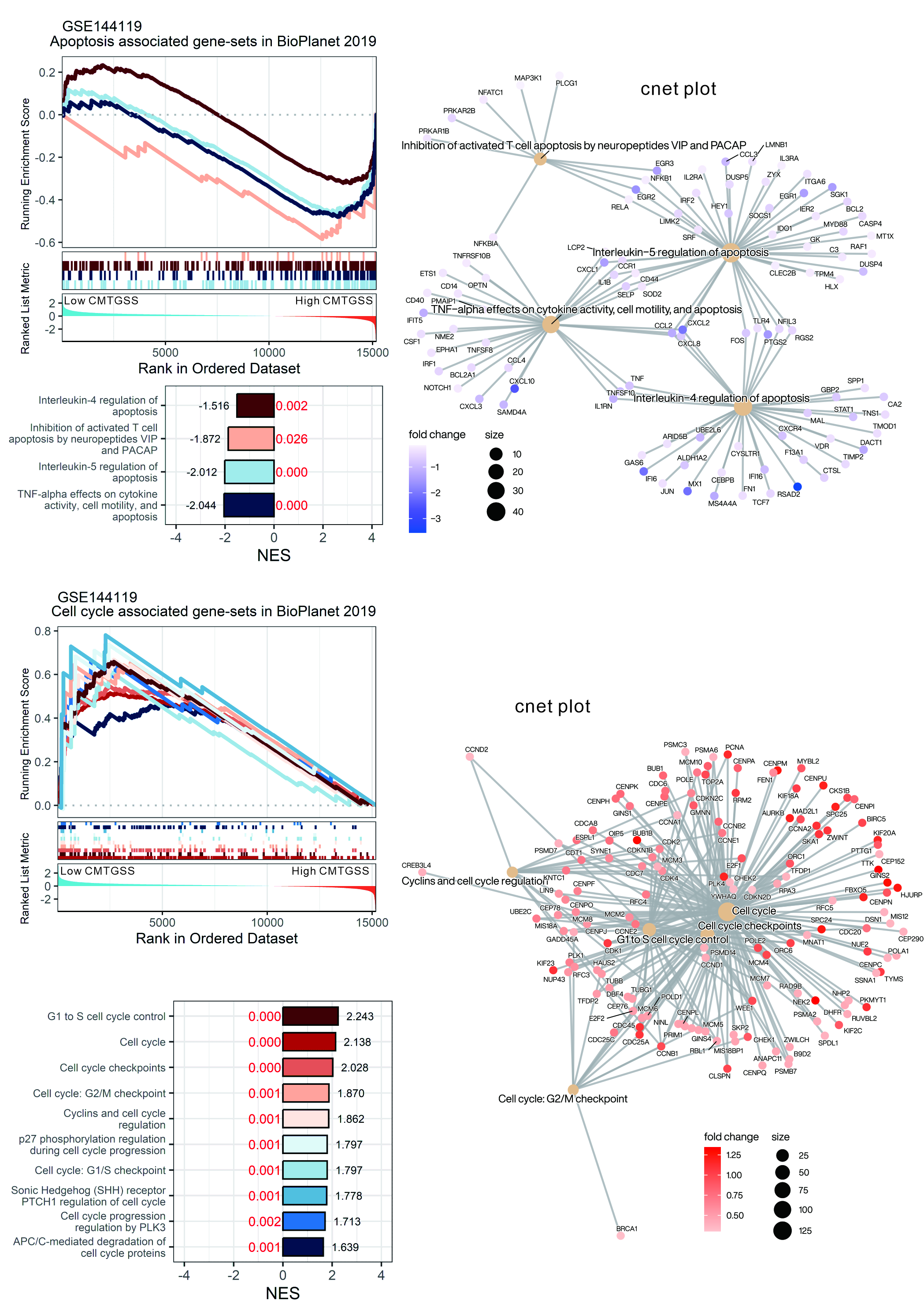

Supplement: Supplementary file 1 [file Image_1.jpeg]
